# Determinants of Maternal Near-Miss in Morocco: Too Late, Too Far, Too Sloppy?

**DOI:** 10.1371/journal.pone.0116675

**Published:** 2015-01-22

**Authors:** Bouchra Assarag, Bruno Dujardin, Alexandre Delamou, Fatima-Zahra Meski, Vincent De Brouwere

**Affiliations:** 1 National School of Public Health, Rabat, Morocco; 2 Department of Public Health, Institute of Tropical Medicine, Antwerp, Belgium; 3 School of Public Health, Université Libre de Bruxelles, Brussels, Belgium; 4 National Centre for Research and Training in Rural Health, Maferinyah, Guinea; Oslo University Hospital, Ullevål, NORWAY

## Abstract

**Background:**

In Morocco, there is little information on the circumstances surrounding maternal near misses. This study aimed to determine the incidence, characteristics, and determinants of maternal near misses in Morocco.

**Method:**

A prospective case-control study was conducted at 3 referral maternity hospitals in the Marrakech region of Morocco between February and July 2012. Near-miss cases included severe hemorrhage, hypertensive disorders, and prolonged obstructed labor. Three unmatched controls were selected for each near-miss case. Three categories of risk factors (sociodemographics, reproductive history, and delays), as well as perinatal outcomes, were assessed, and bivariate and multivariate analyses of the determinants were performed. A sample of 30 near misses and 30 non-near misses was interviewed.

**Results:**

The incidence of near misses was 12‰ of births. Hypertensive disorders during pregnancy (45%) and severe hemorrhage (39%) were the most frequent direct causes of near miss. The main risk factors were illiteracy [OR = 2.35; 95% CI: (1.07–5.15)], lack of antenatal care [OR = 3.97; 95% CI: (1.42–11.09)], complications during pregnancy [OR = 2.81; 95% CI:(1.26–6.29)], and having experienced a first phase delay [OR = 8.71; 95% CI: (3.97–19.12)] and a first phase of third delay [OR = 4.03; 95% CI: (1.75–9.25)]. The main reasons for the first delay were lack of a family authority figure who could make a decision, lack of sufficient financial resources, lack of a vehicle, and fear of health facilities. The majority of near misses demonstrated a third delay with many referrals. The women’s perceptions of the quality of their care highlighted the importance of information, good communication, and attitude.

**Conclusion:**

Women and newborns with serious obstetric complications have a greater chance of successful outcomes if they are immediately directed to a functioning referral hospital and if the providers are responsive.

## Introduction

Between 1990 and 2010, maternal mortality has declined worldwide by nearly 47% [1], and this decline is partly due to better access to skilled care. For many years, the quality of maternal and newborn health was evaluated based on maternal death reviews, but these have limitations related to the reduced number of events and low reliability. These surveys are also limited by the emotions associated with maternal death. Recently, an analysis of cases of severe maternal morbidity referred to as “near misses” (much more common than maternal death) was performed to investigate maternal mortality [2, 3]. A near miss defined by the World Health Organization as “any woman who nearly died but survived a complication during pregnancy, childbirth, or within 42 days of the termination of pregnancy because she received care in health facilities” [4]. A review of near-miss cases highlighted the shortcomings and positive elements of the quality of maternal and newborn healthcare, and these cases of near misses demonstrated similar characteristics to those of maternal death [2,5]. Clinical audit of these cases can help to identify preventable factors that, if addressed, would improve the quality of services offered [6]. A clinical audit also identifies the determinants of near misses and contributes to improving the management of a mother’s severe life-threatening complications [3, 7, 8].

The determinants of near-miss cases are classified into 2 groups of factors: those related to access to care and clinical factors related to quality of care. Geller et al. [7] found that factors related to providers and quality of care are more preventable; these factors are related to delays in healthcare services and influence the state of the mother and newborn at all levels of care demand [7, 9, 10].

In Morocco, few studies have been performed on maternal near misses, and little is known regarding the incidence and determinants of near misses and how to prevent them. The objectives of this study were to determine the incidence of near misses in 3 referral Moroccan hospitals and to identify the avoidable factors that cause obstetric complications leading to near misses.

## Methods

We conducted a case-control study in the districts of Al Haouz and Marrakech from 1 February to 31 July, 2012. These2 districts combined have a population of 1,714,000 inhabitants, and Marrakech is predominantly urban (84%) whereas Al Haouz is predominantly rural (89%). Al Haouz is located approximately 30 km from Marrakech and has an area of 6612 km². This district is characterized by difficult access to healthcare facilities (75% of the area is mountainous), with 83% of the population living 10 km or more from healthcare facilities. These2 study districts contain 20 delivery houses, 3 referral hospitals, and 22 private clinics. The study was conducted simultaneously in the 3 referral maternity hospitals: Mohammed VI Hospital at Al Haouz, the regional Ibn Zohr Hospital in Marrakech, and the University Hospital in Marrakech. Only the University Hospital in Marrakech includes an obstetric intensive care unit (ICU).

### Population

We identified all women who were between the ages of 18 and 49 years, who originated from Marrakech or Al Haouz, who had severe obstetric complications (near misses), and who delivered their children in the 3 study hospitals between 1 February and 31 July, 2012. We applied the definition used by Sahel et al. [11] for screening near miss cases (see S1 Appendix). Sahel et al. [11] combined different criteria, including those based on clinical signs specific for a disease, organ dysfunction, and case management (e.g., admission to an ICU) [12–14]. The controls included women who had the same types of complications as the near misses but who did not reach the stage of near miss. We included complications during pregnancy and childbirth or those within 42 days after delivery. Complications comprised hemorrhage, hypertensive disorders, dystocia, and infection. For inclusion in the study, the controls must have been admitted to the hospital no longer than 48 h after a near miss was identified. We aimed to reduce bias as a result of changes in the care team, and we opted for a ratio of 3 controls per near miss to increase the detection of differences in predictive factors between the cases and controls.

### Procedures

All complicated cases were identified at the end of each week by trained investigators (nurses or midwives) at the 3 hospitals. These cases were reviewed and approved by the principal investigator and the intensive care specialist or the gynecologist. All of the women who were recruited were interviewed in the hospital using a questionnaire administered by the investigators, who collected baseline data on the women’s sociodemographic variables and antenatal, delivery, and postpartum care. Delays in obtaining care were collected according to the 3-delay model [15], which was adapted as follows. (1) Delay at home before deciding to go to a health facility was defined as the number of hours between the onset of labor and the decision to go to a health facility. The source of information was the women, and labor was defined as a set of intensified contractions. (2) Delay in reaching the first health facility was defined as the number of hours between leaving home and reaching the health facility. The source of information was the reference sheet, if it existed, or the woman, her husband, or her family. (3) Delay between the first place of care and the final place of care was divided into 2 phases. The first phase corresponded to the period between arrival at the first facility and arrival at the final one (some women were referred several times). The second phase corresponded to the time spent between arrival at the final facility (last location where the woman was recruited) and the first examination by a midwife or a doctor. The sources of information were the obstetric register and the husband.

We collected information from the respondents regarding the newborns, including gestational age at birth, perinatal mortality or live birth, Apgar scores at 5 and 10 min, and birth weight. The results for the newborns will be published in another article. Information on the women’s history from pregnancy to the postpartum period and on their perceptions of the quality of care was collected from a sample of cases and controls. Each week, we randomly selected 4 women, 2 in the near-miss group and 2 in the control group, to obtain a final sample of 60 women (30 near misses and 30 control women). A specifically trained investigator conducted semi-structured individual interviews with the women at home in Arabic or Berber according to each woman’s preference. The interview mainly consisted of open-ended questions that focused on the women’s perceptions of complications, their experiences with the processes of transfer and care, their opinions and views on the care they received, their contacts with staff, and their suggestions for improving health services. Each interview lasted between 30 and 45 min.

We did not include private clinics in our study because there were none in the Al Haouz district (rural), and the socioeconomic status of the majority of pregnant women did not allow them to attend private clinics [16]. Private clinics in Marrakech are only used by a small number of wealthy women, who are referred to public hospitals in case of severe complications.

The study protocol and consent procedure were approved by the ethics committees of the Institute of Tropical Medicine Antwerp (Belgium), the University of Antwerp, and the University Mohammed V Souissi Rabat (Morocco). The women who participated in the study were informed of the study objectives, and written consents were obtained, documented, and classified. No minor was enrolled in this study.

### Statistical analysis

Statistical analysis was performed using IBM SPSS statistical software, version 20 (New York, USA). The sociodemographic characteristics and descriptions of the near-miss cases and controls were analyzed in 2 stages. First, we compared the proportions of each variable. We then used the chi-square and Fisher’s tests to compare the variations in the proportions among the near misses and controls. Multivariate analysis by logistic regression was used to estimate the association between near miss, low education level, and first and third delays. A p value of 0.05 was considered significant.

Concerning the qualitative component, we analyzed the experiences of women in both groups based on information obtained from the interviews. All of the interviews were transcribed in Arabic and translated into French. The transcripts were analyzed and coded into themes, using the “coding up” method of induction [17], by 2 researchers (the principal investigator and a sociologist) and were analyzed according to thematic content. All of the developed themes were discussed and reported.

## Results

The incidence of near misses in the 3 hospitals was 12 per 1,000 births. Fifty-seven percent of the 80 cases of near misses came from Marrakech, and 43% came from El Haouz.

### Demographics, health, and women’s care

**Sociodemographic characteristics**. Of the 299 women who were enrolled in the study, 80 cases were near misses and 219 were controls. Their mean ages were 29.2 years for the near misses and 28.4 years for the controls. In both the near-miss and control groups, the 20–29 age group was dominant, accounting for 47% and 52% of the participants, respectively. The near-miss group was significantly different from the control group in terms of education and socioeconomic level. The proportion of illiterate women was significantly higher among near-miss cases than controls (65% vs. 22%, p<0.001). A higher proportion of women in the near-miss group belonged to the poorest quintile in comparison to the control group (42% vs. 10%, p<0.001). There was no significant difference between the 2 groups in terms of employment, health insurance, or marital status (Table 1). Only 5 women were single in the studied population, including 3 women who were in the near-miss group.

**Table 1:** Comparison of demographic characteristics.

Characteristics	Near misses, n (%)	Controls, n (%)	p value
Age (years)			
<20	6 (8)	11 (5)	0.50
20–29	38 (47)	114 (52)	
30–39	28 (35)	81 (37)	
≥40	8 (10)	13 (6)	
Unknown	0 (0)	0 (0)	
Total (n)	80 (100)	219 (100)	
Education level			
None	52 (65)	48 (22)	<0.001
Primary	12 (15)	116 (53)	
High school	9 (11)	42 (19)	
Higher education	7 (9)	13 (6)	
Unknown	0	(0)	
Total (n)	80 (100)	219 (100)	
Marital status			
Married	77 (96)	217 (99)	0.12
Single	3 (4)	2 (1)	
Widowed	0 (0)	0 (0)	
Divorced	0 (0)	0 (0)	
Unknown	0 (0)	0 (0)	
Total (n)	80 (100)	219 (100)	
Medical insurance			
Yes	10 (12)	44 (20)	0.18
No	70 (88)	175 (80)	
Unknown	0 (0)	0 (0)	
Total (n)	80 (100)	219 (100)	
Occupation			
Yes	5 (6)	22 (10)	0.24
No	75 (94)	195 (89)	
Unknown	0 (0)	2 (1)	
Total (n)	80 (100)	219 (100)	
Wealth quintiles			
Poorest	34 (42)	22 (10)	<0.001
Second	9 (11)	46 (21)	
Third	8(10)	46 (21)	
Fourth	11(14)	44 (20)	
Wealthiest	10(12)	46 (21)	
Unknown	8 (10)	15 (7)	
Total (n)	80 (100)	219 (100)	

**Obstetric history and pregnancy monitoring**. We did not observe any significant differences in gravidity or parity between the 2 groups. The proportion of a history of abortion was 3 times higher in the near-miss group compared with the controls (21% vs. 7%, p = 0.002). Chronic hypertension was found in 52% of the women in the near-miss group and 47% in the control group. The proportion of diabetes mellitus did not differ between the groups. A higher proportion of women in the near-miss group did not receive follow-up during pregnancy compared to the controls (38% vs. 7%, p<0.001). Additionally, a higher proportion of women in the near-miss group suffered complications during pregnancy compared to the controls (51% vs. 19%, p<0.001) (Table 2).

**Table 2:** Obstetric history and antenatal monitoring.

Characteristics	Near miss n (%)	Controls n (%)	p value
Gravidity			
1	33 (41)	116 (53)	0.10
2–3	33 (41)	81 (37)	
≥4	14 (18)	22 (10)	
Unknown	0 (0)	0 (0)	
Total (n)	80 (100)	219 (100)	
Parity			
1	40 (50)	119 (54)	0.44
2–3	31 (39)	85 (39)	
≥4	9 (11)	15 (7)	
Unknown	0 (0)	0 (0)	
Total (n)	80 (100)	219 (100)	
Abortion			
0	63 (79)	204 (93)	0.002
1	11 (14)	11 (5)	
>2	6 (7)	4 (2)	
Unknown	0 (0)	0 (0)	
Total (n)	80 (100)	219 (100)	
Medical history			
Chronic hypertension	11 (52)	16 (47)	0.06
Diabetes mellitus	8 (38)	6 (17)	
Other	2 (10)	12 (35)	
Total (n)	21 (100)	34 (100)	
Antenatal monitoring			
At least 1 ANC*	50 (62)	204 (93)	<0.001
No	30 (38)	15 (7)	
Unknown	0 (0)	0 (0)	
Total (n)	80 (100)	219 (100)	
Presence of obstetric complications during pregnancy			
Yes	41 (51)	42 (19)	<0.001
No	35 (44)	171 (78)	
Unknown	4 (5)	6 (3)	
Total (n)	80 (100)	219 (100)	

* Antenatal consultation

**Monitoring of delivery time and access to care.** A lower proportion of women (19%) in the near-miss group had access to healthcare facilities within 24 h of the start of labor compared to the controls (67%, p<0.001). The proportion of women in the near-miss group who gave birth at facilities (92%) was lower than that among women in the control group (99%) (Table 3). In addition, a higher proportion of women in the near-miss group took longer than 1 h before reaching the first place of care compared to the controls (37% vs. 6%, p<0.001). We also observed that 83% of women in the near-miss group who took longer than 1 h to reach the final place of care were referred to 1 or 2 healthcare facilities, and the majority of these women came from El Haouz.

**Table 3:** Monitoring of delivery.

Characteristics	Near miss, n (%)	Controls, n (%)	p value
Delay at home before going to a health facility			
≤24 h	15 (19)	147 (67)	<0.001
24–36 h	34 (43)	39 (18)	
>36 h	31 (38)	13 (6)	
Unknown	0 (0)	20 (9)	
Total (n)	80 (100)	219 (100)	
Place of birth			
Home	6 (8)	2 (1)	<0.001
Local home delivery	9 (11)	4 (2)	
Hospital maternity ward	65 (81)	213 (97)	
Unknown	0 (0)	0 (0)	
Total (n)	80 (100)	219 (100)	
Pregnancy outcome			
Vaginal delivery	24 (30)	18 (8)	<0.001
Instrumental	3 (4)	2 (1)	
Caesarean section	53 (66)	199 (91)	
Unknown	0 (0)	0 (0)	
Total (n)	80 (100)	219 (100)	

**Delays and quality of care in health facilities.** A lower proportion of women in the near-miss group received adequate care at the final hospital in less than 30 min compared to the controls (49% vs. 74%, p = 0.001). Among women in the near-miss group who waited more than 1 h before receiving care, 84% were referred from Al Haouz facilities without a referral file, or they were referred directly to the hospital or the maternity ward. A total of 252 (84%) women underwent cesarean sections, and 83% in the near-miss group received a cesarean section in the hour following the decision. All of the women in the near-miss group who had bleeding were transfused, and 72% of these women were transfused in the 15 min following the decision (Table 4).

**Table 4:** Quality of care criteria for women in labor.

Characteristics	Near miss, n (%)	Controls, n (%)	p value
Delay in reaching the final place of care			
<30 min	15 (19)	149 (68)	<0.001
30–60 min	35 (44)	39 (18)	
≥60 min	30 (37)	13 (6)	
Unknown	0 (0)	18 (8)	
Total (n)	80 (100)	219 (100)	
Delay in receiving care at the final place of care			
≤30 min	39 (49)	162 (74)	0.001
30–60 min	23 (29)	18 (8)	
>60 min	15 (18)	13 (6)	
Unknown	3 (4)	26 (12)	
Total (n)	80 (100)	219 (100)	
Delay between the decision to have a caesarean section and the actual operation			
≤30 min	26 (49)	77 (39)	0.84
30–60 min	18 (34)	64 (32)	
>60 min	9 (17)	32 (16)	
Unknown	0 (0)	26 (13)	
Total (n)	53 (100)	199 (100)	
Delay in transfusion (time between demand for blood and blood transfusion)			
≤15 min	41 (72)	7 (44)	
15–30 min	13 (23)	6 (38)	0.24
>30 min	2 (3)	1 (6)	
Unknown	1 (2)	2 (12)	
Total (n)	57 (100)	16 (100)	

**Clinical characteristics of near misses.** Among the 80 cases of near miss, the majority of complications were hypertensive disorders (45%), 26% of these were complicated by hemorrhage and 19% by eclampsia and severe preeclampsia. Bleeding as a result of other causes accounted for 39% of the complications, comprising postpartum hemorrhage (16%), retroplacental hematoma (9%), and placenta previa (8%). Severe infections accounted for 10% of complications and consisted of puerperal septicemia. Dystocia accounted for only 6% of complications, including uterine (4%) and pre-uterine ruptures (2%) (Table 5).

**Table 5:** Distribution of near-miss complications.

Categories of complications*		Cases of near miss	
		n	%
Hemorrhage		31	39
	Hemorrhagic shock	2	3
	Postpartum hemorrhage	13	16
	Disseminated intravascular coagulation	1	1
	Placenta previa	6	8
	HELLP syndrome	1	1
	Placental abruption	7	9
	Uterine inertia	1	1
Hypertensive disorders		36	45
	Preeclampsia and eclampsia complicated by bleeding	21	26
	Preeclampsia	7	9
	Eclampsia	8	10
Dystocia		5	6
	Pre-uterine rupture	2	2
	Uterine rupture	3	4
Infection		8	10
	Sepsis	8	10
Organ dysfunction		4	5
	Acute pulmonary edema	1	1
	Diabetic coma	1	1
	Heart disease	2	3

*Near misses may have multiple complications.

### Determinants of near misses

We investigated the factors that explained the risk of near miss in women with complications. These factors were grouped into 3 categories: socioeconomic and demographic factors, factors related to the women’s obstetric histories, and factors related to time management (Table 6).

**Table 6:** Analysis of near-miss determinants by binary logistic regression.

Variables	Univariate analysis OR(95% CI)	Multivariate analysis OR (95% CI)
Age (years)		
<20	1.70 (0.60–4.84)	-------
20–34	1	
≥35	1.44 (0.81–2.57)	-------
Education level		
None	6.62 (3.78–11.58)	2.35 (1.07–5.15)
Educated	1	1
Wealth quintiles		
Poor (poorest/second poorest)	2.97 (1.71–5.16)	-------
Not poor (others)	1	
Abortion		
0	1	
≥1	3.67 (1.73–7.77)	-------
Antenatal monitoring		
Yes	1	1
No	8.16 (4.08–16.31)	3.97 (1.42–11.09)
Presence of obstetric complications during pregnancy		
Yes	4.77 (2.72–8.38)	2.81 (1.26–6.29)
No	1	1
Delay in reaching the first place of care (at home)		
≤24 h	1	1
>24 h	12.25 (6.43–23.33)	8.71 (3.97–19.12)
Delay in reaching the final place of care		
<60 min	1	1
≥60 min	12.42 (6.52–23.64)	4.03 (1.75–9.25)
Delay in receiving care at the final place of care		
<60 min	1	--------
≥60 min	3.35 (1.51–7.43)	--------

Considering only the sociodemographic factors in our analysis, we observed that low education level and low socioeconomic status were associated with near misses. With regard to obstetric variables, women who did not receive antenatal care were 8 times more likely to be near-miss cases [OR = 8.16; 95% CI: (4.08–16.31). Those with complications during pregnancy had a 4-fold higher risk [OR = 4.77; 95% CI: (2.72–8.38) and those with a history of abortion had a 3-fold higher risk for complication [OR = 3.67; 95% CI: (1.73–7.77). These results were supported by individual interviews with the women, providing responses such as “I did not attend antenatal care because the center is too far”; “I don’t live in Marrakech where the doctor is close by”; “and I felt good…but then I almost died…God has saved me” (26 years old, near miss, El Haouz).

We also examined the effect of time management variables using an intermediate model that included only delays. All variables related to time were significantly associated with near misses. The risk of near miss was higher in women who went into labor >24 h before visiting a health facility [OR = 8.71; 95% CI (3.97–19.12)] and 4 times higher in women who took ≥ 60 min before reaching the final facility [OR = 4.03; 95% CI (1.75–9.25)]. Women who had a delay of ≥60 min in receiving care at the final facility had a 3-fold higher risk of near miss [OR = 3.35; 95% CI: (1.51–7.43)]. All significant variables in the 3 intermediate models described above were included in the final model. Table 6 shows the final model after the step-by-step exclusion of variables that became non-significant after adjustment for other variables. This model included age, education level, socioeconomic status, history of abortion, antenatal care, obstetric complications during pregnancy, duration of labor before reaching the place of care, time to arrive at the final facility, and time management at the place of recruitment.

Low education level, lack of antenatal care, presence of obstetric complications during pregnancy, labor duration >24 h before reaching a healthcare facility, and time to reach the final place of care >1h were the main determinants of near misses in our sample (.

The main reasons given by the women for the first delay (time at home before visiting a health facility) were lack of a parent’s authority to go to a facility (10 cases) and fear of the health facility (12 cases). The women in roughly one-third of near-miss cases in El Haouz had attempted delivery assisted by a traditional birth attendant.

With regard to the second delay (delays caused by the women before they reached the first place of care), lack of financial resources (8 cases) and lack of a vehicle (3 cases) were the main factors that delayed access to emergency obstetric care.

For the third delay (delay between the first health facility accessed and the place where care was actually provided), we considered 2 categories: (1) the experiences of the women and their families with the intermediate facilities, including multiple referrals after reaching a health facility, and (2) delays in care after arrival at the final place of care. Multiple referrals were recorded in 21 of the 30 near misses. Eight of these 21 women developed complications at home, and 13 developed complications in a healthcare facility. These women had all made several trips between the health center and the peripheral maternity ward or the hospital and a university hospital. They also received care only after a delay of 4 h (although the time to reach the university hospital was at most 1 h). Seventy-five percent of women interviewed were satisfied with the quality of care in the ICU, and 25% of the near misses appreciated the behavior of their midwives: “*The midwife was good*, *she told me everything that I needed to do*. *I could not feel my baby move*, *but she supported me…God bless her*…” (32 years old, near miss, stillborn, El Haouz).

In contrast, nearly all of the women (86%) reported dissatisfaction with their providers’ behavior once they were transferred to postpartum service. Others reported a delay in obtaining their free services and a lack of adequate information on their health: “*They did not tell me anything*. *So after approximately 3 days*, *I said that ‘nobody has told me anything’*. *My sister answered my questions after the hysterectomy*. *I haven’t even seen my baby for 2 days*” (33 years old, near-miss, live birth, El Haouz).

## Discussion

### Incidence and determinants of near misses

The incidence of near misses in our study was 12‰ among hospital deliveries, which is similar to that observed in Europe. Van Roosmalen et al. [18] observed that this incidence was in the range of 3.8–12% in developed countries. In a study of 9 hospitals in West Africa and Morocco, Filippi et al. [19] observed that the incidence of near misses was in the range of 11.7–237.5 per 1,000 hospital births. In addition, Adisasmita et al. [20] reported near-miss incidence rates between 4.2% and 17.3% in 4 hospitals in Indonesia. This variation could be attributable to the differences in definitions used by researchers, or it could reflect the true epidemiological reality. Standardization of the definition of maternal near miss would better describe the reality of this phenomenon in countries with limited resources. Thus, these countries need to carefully identify near misses to prioritize interventions and to improve the quality of maternal and neonatal health services.

In our study, the incidence of near misses was twice as high in rural areas (20‰) compared to urban areas (10‰). The risks associated with pregnancy and childbirth and the circumstances of timely access to care differed between women from El Haouz and Marrakech, and these differences are most likely related to socioeconomic status, education level, and the level of isolation in El Haouz. Among the cases of near miss, hemorrhage and hypertensive disorders were the main complications observed. This finding is consistent with those of studies in most developing countries [21–23], and these obstetric events are also the main causes of maternal death in Morocco [24]. In addition, first and third delays were determinants of near misses in our study, and multiple referrals also had a considerable effect in delaying access to quality obstetric care.

### Sociodemographic and obstetric determinants of near misses

In our study, a low level of education was the only factor associated with the occurrence of near misses among the sociodemographic and obstetric characteristics after adjusting for all other factors. The majority of studies published to date have confirmed this association between education level and maternal near misses [25, 26].

### Delays in obtaining care

Delaying the decision to seek care was a significant risk factor that contributed to maternal near misses. Even though some of the women had experienced complications during pregnancy (51% of near misses and 19% of controls), 10 near-miss patients from El Haouz had attempted delivery at home assisted by a traditional birth attendant, and 38% of near-miss patients had no antenatal care. This first delay was a direct consequence of how the woman weighed the risks [27–29]. During the interviews, the women reported that the inability to pay for transport and fear of hospitals were the factors that forced them to bear the pain instead of going to the hospital. Other studies have shown that living conditions, financial difficulties, and lack of physical access to a health facility are predictors of morbidity and maternal and neonatal mortality [30–34].

With regard to the third delay, multiple factors contributed to these delays. In our study, women who took ≥60 min to reach the final facility were 4 times more likely to be near misses. The majority of these women reported more difficulties in physical access to a health facility (second delay). Additionally, once they were in a facility, they were transferred from one facility to another without receiving any information. These delays in peripheral maternity wards, travel, and multiple referrals from one facility to another led to poorer outcomes for the health of these women and/or their newborns. A number of studies have shown that various factors related to health systems play an important role in maternal and neonatal complications [34–37]. These factors include confusion regarding professional responsibilities, inadequate equipment, and insufficient human resources (number and competence of providers) [21, 35, 38]. The absence of clear guidelines and the lack of support from decision makers could have affected providers’ confidence and resulted in their refusals to take risks with women with complications. Indeed, the women reported that their healthcare providers did not provide sufficient communication, and providers are often judged by the community in relation to their unfriendly manner with women [39, 40].

With regard to support at the final place of care, all of the women reported being grateful for the care they had received, and they stressed that they would not have survived if they had not been treated in intensive care. The women’s perceptions of the quality of their care highlighted a number of important factors: the importance of information, good communication, and positive provider attitudes and the availability of human resources and equipment. Quality care should begin prenatally and should provide women with information about their deliveries. Some husbands noted that they were unaware of the importance of the referral sheet that allows them to have free care at the right time, especially in the hospitals. The women reported their wish to ask questions and obtain clear answers. However, for many women in our study, this was not possible because either they were in intensive care or they were afraid to speak. Indeed, previous studies have reported the importance of reassuring women and addressing their concerns and safety [17, 39, 41, 42].

### Strengths and limitations of the study

Our study is the first to address the determinants of maternal near misses in relation to the quality of obstetric care in Morocco. We studied women’s experiences and the timing of their care by health professionals. One primary strength of our study is that it was prospective; details on the circumstances of care from home to the final healthcare facility were still fresh in the women’s memories. In addition, the use of multiple sources of information (registers/hospital records, reports on medical circumstances, and interviews with women) represented another strength of our study.

However, our study had limitations. The selection of women was limited to those who reached the hospital, and we did not analyze the data from each health facility. Therefore, we could not perform an analysis according to the type of institution or according to the availability of equipment, which are both determinants of quality of care. Finally, we recorded only the women’s perceptions but not the healthcare personnel’s opinions.

## Conclusion

Our study showed that the occurrence of maternal near misses was associated with women’s low education levels, delays in the decision to go to a healthcare facility, and delays in receiving care at the first healthcare facility visited. Health centers and peripheral maternity wards contributed to these delays, and it is therefore important to promptly address the causes for these delays. The attitudes of peripheral-level health personnel are known to be a determinant in increasing the population’s trust and in motivating women to deliver in healthcare facilities. Moreover, a respectful attitude is not sufficient—healthcare personnel should have high levels of competence and the appropriate equipment to manage complications that require emergency obstetric care, which is the responsibility of the health system managers.

## Supporting Information

S1 Appendix Sahel et al: Criteria for maternal near miss. (DOCX)
