# Supplementary material for: Determinants of Maternal Near-Miss in Morocco: Too Late, Too Far, Too Sloppy?
Source: PLoS One. 2015 Jan 22;10(1):e0116675. doi: 10.1371/journal.pone.0116675 (PMC4303272; doi:10.1371/journal.pone.0116675)
Supplement: S1 Appendix — (DOCX) [file pone.0116675.s001.docx]

**Appendix S1.** Sahel & al: Criteria for maternal near miss

| Severe preeclampsia and eclampsia | (a) Blood pressure >140/90 mmHg or a change in systolic blood pressure >30 mmHg or diastolic blood pressure >15 mmHg AND at least 1 of the following: convulsions, coma, jaundice, pulmonary edema, severe oliguria, massive proteinuria, thrombocytopenia <100,000 platelets, eclampsia (at least 2 of the following symptoms: blurred vision, frontal headache, and epigastric bar) or(b)Seizures AND antihypertensive therapy or massive proteinuria or generalized edema |
| --- | --- |
| Severe infection (a) and septic shock (b) | (a) Clinical diagnosis of sepsis mentioned in the file or (b) Temperature >39 or <36°C or genitourinary infection AND one of the following signs: systolic blood pressure of 80 mmHg, jaundice, impaired consciousness, or oliguria <100 mL in 4 h |
| Disseminated intravascular coagulation | All |
| Hysterectomy | All |
| Placental abruption | All |
| Uterine rupture or pre-uterine rupture | All |
| Coma | All |
| Shock | (a) Systolic blood pressure ≤60 mmHg or (b) systolic blood pressure ≤80 mmHg and 2 of the following symptoms: sweating, thready and rapid pulse, pallor, cyanosis, and cold extremities, or oliguria <100 mL in 4 h, or (c) at least 2 units of blood or 2 L of saline perfused in 1 h (fast fill). |
| Acute pulmonary edema | All |

Source: Sahel & al. [11]
